# Use of dupilumab in the management of refractory prurigo nodularis in an immunosuppressed double transplanted patient^؆^

**DOI:** 10.1016/j.abd.2026.501358

**Published:** 2026-05-06

**Authors:** Diego Valenzuela Godoy, Rosario Agüero, Marianne Kolbach

**Affiliations:** a Faculty of Medicine, Universidad de los Andes, Santiago, Chile; b Department of Dermatology, Faculty of Medicine, Universidad de los Andes, Santiago, Chile; c Department of Dermatology, Clínica Universidad de los Andes, Santiago, Chile

Dear Editor,

Prurigo nodularis is a chronic inflammatory dermatosis characterized by intensely pruritic nodules. Its pathophysiology involves a complex neuro-immunological interaction, with a Th2-type inflammatory response and neurogenic mediators such as substance P.^1^, ^2^ Its management is complex, and conventional treatments are often insufficient to control pruritus and lesions.^3^

There is evidence that immunosuppression, whether due to underlying diseases or specific treatments, may be associated with the onset of prurigo nodularis, although the relationship is neither exclusive nor pathognomonic.^1^ However, while there are new therapies for nodular prurigo, including the use of dupilumab, immunosuppressed patients have been systematically excluded from clinical trials evaluating them.

We present the case of a double transplant recipient and immunosuppressed patient with refractory prurigo nodularis who was successfully treated with dupilumab.

A 71-year-old male patient with a history of type 1 diabetes mellitus and chronic kidney disease due to diabetic nephropathy. He underwent a double kidney and pancreas transplant and has been on immunosuppressive treatment since 2005, currently with tacrolimus 3 mg every 12-hs and prednisone 10 mg every 24-hs.

His last hospitalization was due to septic shock of gastrointestinal origin, where he developed a purpuric vesicular-bullous rash predominantly on the trunk and extremities, consistent with septic vasculitis due to *Streptococcus pyogenes* with skin involvement. An antibiotic regimen with ceftriaxone was completed, and multiple surgical cleanings and escharectomies were performed for necrotic lesions. After resolution of the condition, the patient was discharged with dermatology follow-up.

One month after discharge, well-defined, pruritic, rounded erythematous skin lesions were observed on the right shoulder and back, consistent with nummular eczema (Fig. 1). General measures and treatment with topical corticosteroids and antibiotics were indicated. One month later, the lesions became more hypertrophic and widespread, involving the abdomen and chest, associated with intense pruritus that interrupted sleep. Given the progression of the condition, systemic therapy with prednisone was initiated, and a skin biopsy was requested, which revealed drug-induced spongiotic dermatitis.

**Figure 1:**
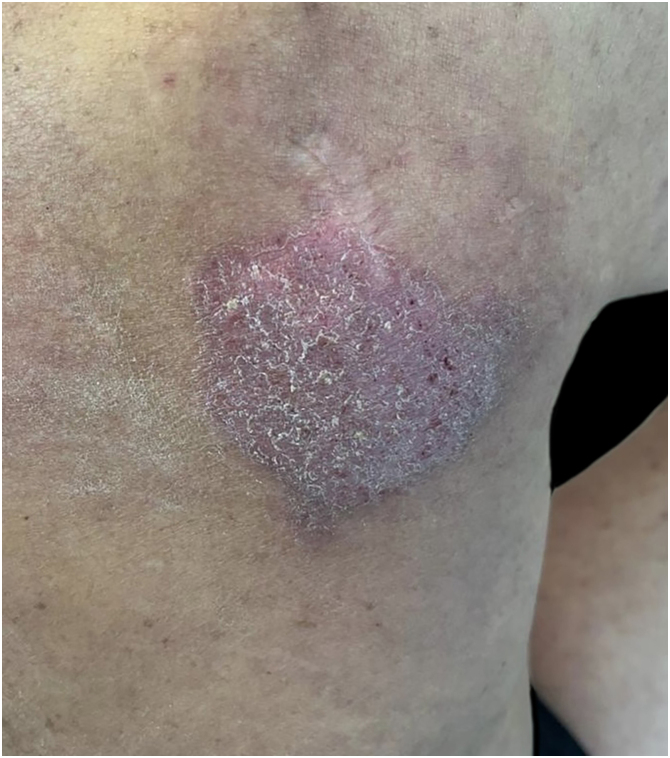
Rounded, erythematous, eczematous plaques consistent with nummular eczema.

He subsequently continued with partial improvement of symptoms, with the addition of indurated erythematous-violaceous nodules on the anterior aspect of both arms (Fig. 2). A diagnosis of nodular prurigo associated with severe, extensive nummular eczema was established, and therapy was adjusted. Despite multiple topical and systemic therapeutic strategies and phototherapy, he persisted without clinical improvement.

**Figure 2:**
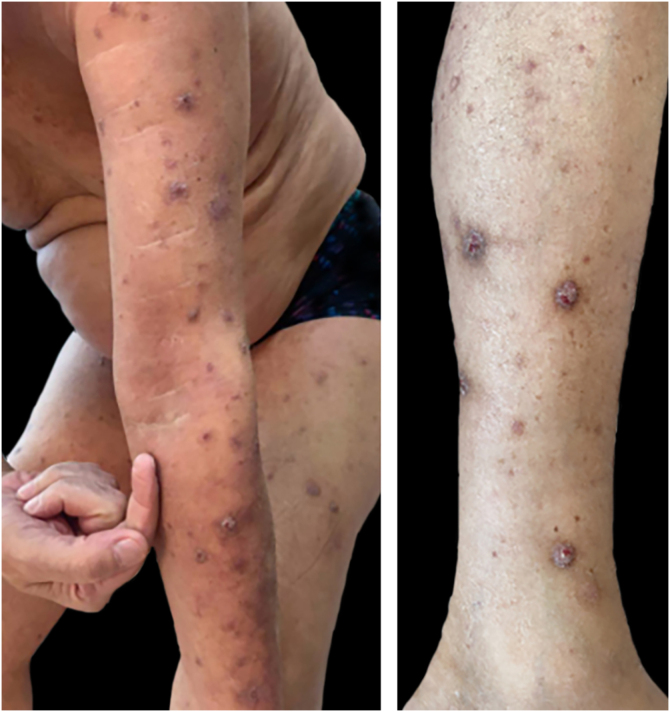
Prurigo nodularis. Indurated, erythematous-violaceous nodules on the anterior aspect of both arms and legs.

Given the difficulty in managing the condition due to the patient's history of double transplantation and immunosuppression, treatment with dupilumab was initiated. At the time of the last check-up, after 8 injections (4-months of treatment), a good response was reported, with a decrease in the number of active lesions (Fig. 3) from 30 to 7, relief of pruritus to 2/10, and improvement in nighttime sleep. Thus, biological treatment proved to be effective in controlling the disease, significantly improving the patient's quality of life.

**Figure 3:**
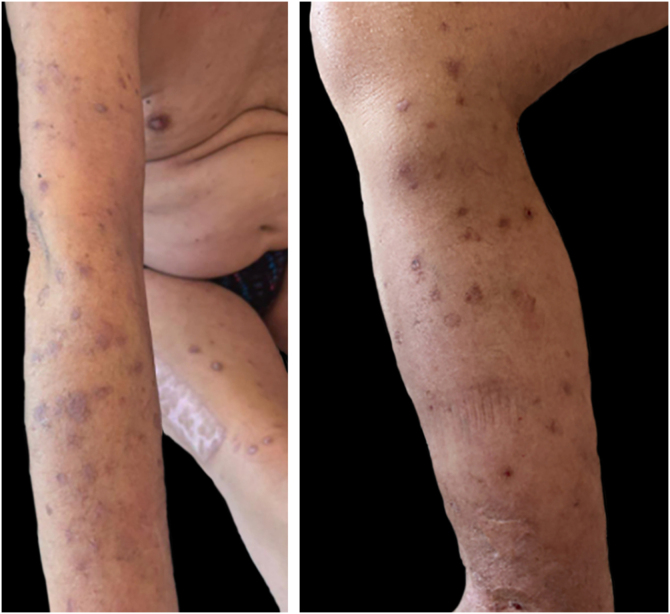
Skin lesions after 4-months of treatment with dupilumab.

The case presented illustrates the complexity of managing prurigo nodularis in a patient with multiple comorbidities and coexisting dermatological conditions, which hinder treatment and remission. This complexity is accentuated in the context of organ transplantation, where nodular prurigo may be related to both immunosuppression and additional risk factors, such as chronic infections or host-specific immunological alterations.^4^

Kidney transplant patients, especially those with end-stage kidney disease or transplantation, are particularly susceptible, with the severity of renal disease being an independent and significant risk factor for the development of prurigo nodularis.^5^

In this scenario, dupilumab has emerged as an effective therapeutic alternative, blocking the IL-4/IL-13 pathway, reducing both pruritus and the number and severity of nodular lesions, positioning itself as the treatment of choice in cases refractory to conventional therapies.^6^ Clinical trials have demonstrated its efficacy and safety, with no relevant increase in serious infections or major adverse events.^7^ However, these studies systematically excluded patients with significant immunosuppression, including solid organ transplant recipients, so evidence in this group is limited to case reports and small series.^4^, ^7^

The reviewed literature suggests that dupilumab could be a valid therapeutic option in patients with prurigo nodularis and immunosuppression, due to its targeted mechanism of action and safety profile that does not cause global immunosuppression, unlike other classic immunomodulators.^4^, ^7^ Moreover, drug interactions are almost absent, making it suitable for elderly patients with comorbidities and polypharmacy.^8^

## ORCID IDs

Diego Valenzuela Godoy: 0009-0006-0799-2008

Rosario Agüero: 0000-0001-5476-8494

Marianne Kolbach: 0009-0002-1465-1085

## Research data availability

Does not apply.

## Financial support

This research did not receive any specific grant from funding agencies in the public, commercial, or not-for-profit sectors.

## Authors' contributions

Diego Valenzuela Godoy: The study concept and design, data collection, or analysis and interpretation of data, writing of the manuscript or critical review of important intellectual content, effective participation in the research guidance, intellectual participation in the propaedeutic and/or therapeutic conduct of the studied cases, critical review of the literature and final approval of the final version of the manuscript.

Rosario Agüero: The study concept and design, data collection, analysis and interpretation of data, writing of the manuscript or critical review of important intellectual content, effective participation in the research guidance, intellectual participation in the propaedeutic and/or therapeutic conduct of the studied cases, critical review of the literature, and final approval of the final version of the manuscript.

Marianne Kolbach: The study concept and design, data collection, analysis and interpretation of data, writing of the manuscript, or critical review of important intellectual content, effective participation in the research guidance, intellectual participation in the propaedeutic and/or therapeutic conduct of the studied cases, critical review of the literature and final approval of the final version of the manuscript.

## Conflicts of interest

None declared.
